# Dietary fructose and risk of metabolic syndrome in adults: Tehran Lipid and Glucose study

**DOI:** 10.1186/1743-7075-8-50

**Published:** 2011-07-12

**Authors:** Firoozeh Hosseini-Esfahani, Zahra Bahadoran, Parvin Mirmiran, Somayeh Hosseinpour-Niazi, Farhad Hosseinpanah, Fereidoun Azizi

**Affiliations:** 1 Obesity Research Center, Research Institute for Endocrine Sciences, Shahid Beheshti University of Medical Sciences. No 24 Parvaneh St, Yemen St, Chamran Exp, Tehran, 19395-4763, Iran; 2 Department of Clinical Nutrition and Dietetics, Faculty of Nutrition Sciences and Food Technology, National Nutrition and Food Technology Research Institute, Shahid Beheshti University of Medical Sciences. No 46 Arghavan-e-gharbi St, Farahzadi Blv, Shahrak-e-Ghods. Tehran, 19395-4741, Iran; 3 Endocrine Research Center, Research Institute for Endocrine Sciences, Shahid Beheshti University of Medical Sciences. No 24 Parvaneh St, Yemen St, Chamran Exp, Tehran, 19395-4763, Iran

**Keywords:** Dietary fructose, Metabolic syndrome, Tehran Lipid and Glucose Study

## Abstract

**Background:**

Studies have shown that the excessive fructose intake may induce adverse metabolic effects. There is no direct evidence from epidemiological studies to clarify the association between usual amounts of fructose intake and the metabolic syndrome.

**Objective:**

The aim this study was to determine the association of fructose intake and prevalence of metabolic syndrome (MetS) and its components in Tehranian adults.

**Methods:**

This cross-sectional population based study was conducted on 2537 subjects (45% men) aged 19-70 y, participants of the Tehran Lipid and Glucose Study (2006-2008). Dietary data were collected using a validated 168 item semi-quantitative food frequency questionnaire. Dietary fructose intake was calculated by sum of natural fructose (NF) in fruits and vegetables and added fructose (AF) in commercial foods. MetS was defined according to the modified NCEP ATP III for Iranian adults.

**Results:**

The mean ages of men and women were 40.5 ± 13.6 and 38.6 ± 12.8 years, respectively. Mean total dietary fructose intakes were 46.5 ± 24.5 (NF: 19.6 ± 10.7 and AF: 26.9 ± 13.9) and 37.3 ± 24.2 g/d (NF: 18.6 ± 10.5 and AF: 18.7 ± 13.6) in men and women, respectively. Compared with those in the lowest quartile of fructose intakes, men and women in the highest quartile, respectively, had 33% (95% CI, 1.15-1.47) and 20% (95% CI, 1.09-1.27) higher risk of the metabolic syndrome; 39% (CI, 1.16-1.63) and 20% (CI, 1.07-1.27) higher risk of abdominal obesity; 11% (CI, 1.02-1.17) and 9% (CI, 1.02-1.14) higher risk of hypertension; and 9% (CI, 1-1.15) and 9% (1.04-1.12) higher risk of impaired fasting glucose.

**Conclusion:**

Higher consumption of dietary fructose may have adverse metabolic effects.

## Introduction

Fructose is the sweetest tasting carbohydrate, found in many fruits and vegetables. In the past, dietary intake of fructose was used to be 16-20 grams per day, mainly from fresh fruits and vegetables. But in the last three decades, increased consumption of industrialized foods such as soft drinks, fruit juices, bakery products, canned fruits, jams, jellies and cookies, containing added sugars (sucrose, high fructose corn syrup, honey, molasses, and other syrups) has resulted in a significant increase in fructose intakes of 85-100 grams per day [1,2]. Recent data suggest parallel increasing trends in fructose intake and the increase in obesity and type 2 diabetes in the last 35 years [3,4]. Reports confirmed by animal and human clinical studies, indicating that the excessive fructose intakes induce adverse metabolic effects [5-10]; however there is no direct evidence from epidemiological studies to clarify the association between current amounts of dietary fructose intake and the metabolic syndrome components. Metabolic syndrome (MetS), a worldwide epidemic health problem, is characterized by central obesity, hypertension, insulin resistance, and lipid profiles abnormalities [11]. The prevalence of MetS in Iranian adults is reported to be one of the highest worldwide, with a rate of 33.7% [12,13]. Considering the lack of data on dietary fructose intake in Iranian adults, the aim of this cross sectional study was to assess dietary intakes of fructose and to investigate the association between fructose intake and prevalence of MetS risk factors, in a sample selected from the Tehran Lipid and Glucose Study.

## Methods

### Participants

Data were obtained from subjects of the Tehran Lipid and Glucose Study (TLGS) between 2006-2008. Details of the Tehran Lipid and Glucose Study have been reported elsewhere [14,15]. Briefly, TLGS is a community-based prospective study conducted to investigate and prevent non-communicable diseases, in a representative sample of residents, aged ≥ 3y, from district 13 of Tehran, the capital of Iran. The first phase of the TLGS began in March 1999 and data collection, at three-year intervals, is ongoing [15]. From 12 523 subjects, aged ≥ 3 years, in 2006-2008, 4920 were randomly selected for dietary assessment, based on age and sex categorization, and 3462 (70 percent) completed the dietary assessment [16]. Of these, 2799 men and women aged 19-70 y, were recruited for this study. Subjects with under- or over reporting of dietary intakes (less than 800 kcal/d or more than 4200 kcal/d, respectively) were excluded. Finally, the data of 2537 adults (1141men and 1396 women) were analyzed.

### Dietary intake assessment and fructose intake estimation

Dietary data were collected by using a validated semi-quantitative food frequency questionnaire (FFQ) with 168 food items, based on consumption frequency for each food item during the past year, on a daily, weekly or monthly basis [17]. Since the Iranian Food composition Table (FCT), with limited data on nutrient content of raw foods and beverages, is incomplete, the U.S Department of Agriculture (USDA) FCT was used to calculate energy and nutrient intakes [18]. However the Iranian FCT was used for some national foods that are not listed in the USDA FCT [19]. Dietary intakes of naturally-occurring fructose from fructose-containing food such as fruits, vegetables, honey, etc, were defined as "natural fructose (NF)" and were calculated using the USDA FCT. Dietary simple carbohydrates was defined as total intakes of disaccharides (sucrose, galactose, lactose), monosaccharides (fructose, glucose, maltose), and the content of added sugar in industrialized foods. Intakes of fructose from industrialized foods and beverages containing beet or cane sugar/molasses, corn sweeteners and invert syrup were defined as "added fructose (AF)". Since there are no databases for added sugar content of Iranian food products, the USDA database for added sugar was used to identify the added sugar contents of food items [20,21]. The most commonly added sugar in Iranian food products is sucrose, while other sweeteners such as corn syrup are not commonly employed. Hence 50% of added sugar in food products was considered as fructose. Eventually the intakes of total fructose were calculated by summing up natural fructose and added fructose consumed.

### Clinical and biological measurements

Trained interviewers collected information using a pretested questionnaire [14]. Weight was measured to the nearest 100 g with digital scales, while the subjects were minimally clothed without shoes. Height was measured to the nearest 0.5 cm, in a standing position without shoes, using a tape meter. Waist circumference was measured to the nearest 0.1 cm, at the umbilical level and that of the hip, at the maximum level, over light clothing, using an unstretched tape meter, without any pressure to the body. Body mass index was calculated as weight (kg) divided by square of the height (m^2^). For blood pressure measurements, after a 15-minute rest in the sitting position, two measurements of blood pressure were taken, on the right arm, using a standardized mercury sphygmomanometer; the mean of the two measurements was considered as the participant's blood pressure. Fasting blood samples were taken after 12-14 h, from all study participants. Fasting plasma glucose was measured by the enzymatic colorimetric method using glucose oxidase. Triglyceride levels were measured by enzymatic colorimetric analysis with glycerol phosphate oxidase. High-density lipoprotein cholesterol (HDL-C) was measured after precipitation of the apolipoprotein B containing lipoproteins with phosphotungistic acid. Analyses were performed using Pars Azmon kits (Pars Azmon Inc., Tehran, Iran) and a Selectra 2 auto-analyzer (Vital Scientific, Spankeren, Netherlands). Inter- and intra assay coefficient of variation of all assays were < 5% [14].

### Metabolic syndrome definition

Metabolic syndrome was defined according to the diagnostic criteria proposed by NCEP ATP III, and new cutoff points of waist circumference for Iranian adults; it was characterized as having at least 3 of the metabolic abnormalities: 1) Fasting plasma glucose ≥ 100 mg/dL (5.6 mmol/L) or drug treatment of hyperglycemia, 2) serum triglycerides ≥ 150 mg/dL (1.69 mmol/L) or drug treatment, 3) serum HDL-cholesterol < 40 mg/dL (1.04 mmol/L) for men, and < 50 mg/dL (1.29 mmol/L) for women or drug treatment, 4) blood pressure ≥ 130/85 mmHg or drug treatment for hypertension, and 5) waist circumference ≥ 95 cm for both sexes [22,23].

### Statistical analysis

Statistical analysis was performed using SPSS (Version 16.0; Chicago, IL), with *P *values < 0.05 being considered significant. Dietary intakes of fructose were adjusted for energy intakes: [(fructose intake (g/d) × 1000/energy intake (kcal/d)]. Total dietary fructose was assigned as quartile intakes for men and women, based on their 25^th^- 50^th^- 75^th ^percentile values. Total dietary fructose intakes (g/1000 kcal/d) in the 1^st^, 2 ^nd^, 3^rd ^and 4^th ^quartiles were ≤ 13.3, 13.4-17.5, 17.6-23.7, and > 23.7 in men, and ≤ 10.3, 10.4-15.2, 15.3-20.8, and > 20.8 in women. Differences in general characteristics of participants across quartiles of fructose intakes were compared using one-way analysis of variance or the Chi-square test. Age adjusted means for dietary intakes across quartiles of fructose, were determined by using the general linear model. Means of metabolic syndrome features were compared across quartiles of fructose intake by using general linear model with adjustment for age (categorized, 19-30, 31-40, 41-50, 51-60, 61-70; groups 1 to 5), physical activity (continuous), energy intake (kcal/d), and smoking (yes or no). The associations between total dietary fructose and metabolic syndrome risk factors, as continuous variables, were determined by using multivariate linear regression model with adjustment for age (categorized), BMI (except for waist circumference and MetS), physical activity (in MET-h/wk), smoking (yes or no), energy intake (kcal/d), percentage of energy from fat, carbohydrate and simple carbohydrate (except fructose), total fiber intake (g/d), and also adjustment for current estrogen use (yes or no) and menopausal status (yes or no) in women. Also, the odds ratio of MetS and its component in each quartiles of total fructose intake was determined by using multivariable logistic regression model with adjustment for the above-mentioned confounders. Since, the odds ratio, which was estimated from logistic regression models in cross sectional studies, is not a valid estimation in binary outcome variables with a high prevalence (> 10%), the adjusted odds ratios were corrected by the formula suggested by Zhang and Yu, for better estimation of the association [24,25]. To assess the overall trends of odds ratios across increasing quartiles of total fructose intake, the median fructose intake of each quartile was used as a continuous variable in logistic regression models [26].

## Results

**Figure 1:**
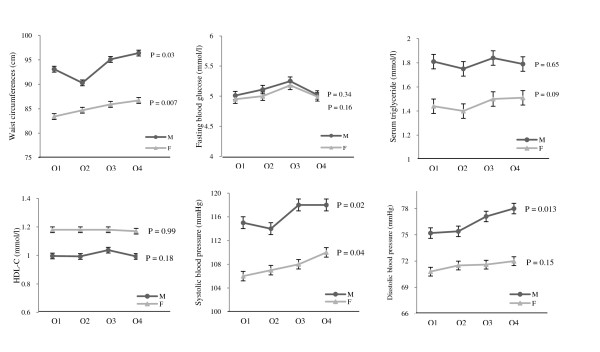
**The means of metabolic syndrome components across quartiles of fructose intakes**. General linear models were used with adjustment for age, physical activity, body mass index, smoking status, energy intake, dietary intake of carbohydrate, fat, simple carbohydrate (except fructose),and fiber, and additionally adjusted for current estrogen use and menopausal status in women. Total dietary fructose intakes (g/1000 kcal/d) in 1^th^, 2 ^th^, 3^th ^and 4^th ^quartiles were ≤13.3, 13.4-17.5, 17.6-23.7, and > 23.7 in men, and ≤ 10.3, 10.4-15.2, 15.3-20.8, and > 20.8 in women. Dietary fructose intake positively associated with waist circumference and systolic blood pressure in both sexes and diastolic blood pressure in men.

**Figure 2:**
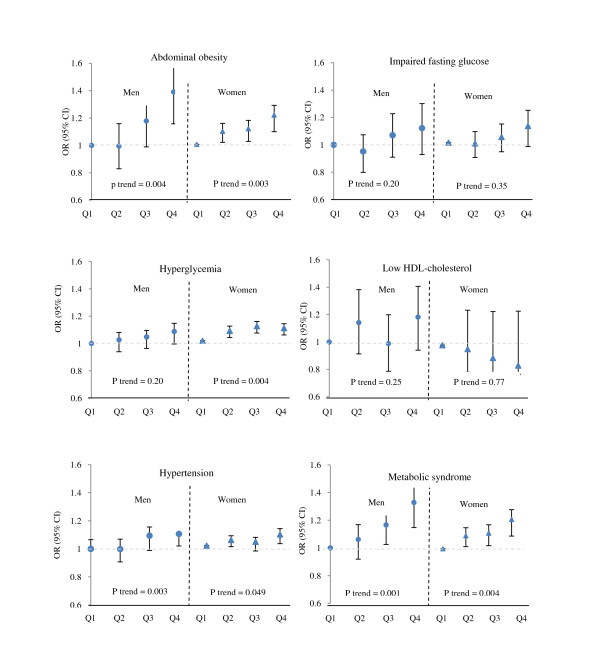
**Multivariate odds ratio and 95% confidence interval for the metabolic syndrome and its components across quartiles of fructose intake**. Logistic regression models were used with adjustment for age, physical activity, body mass index, smoking status, energy intake, dietary intake of carbohydrate, fat, simple carbohydrate (except fructose), fiber, and additionally adjusted for current estrogen use and menopausal status in women. Total dietary fructose intakes (g/1000 kcal/d) in 1^th^, 2 ^th^, 3^th ^and 4^th ^quartiles were ≤ 13.3, 13.4-17.5, 17.6-23.7, and > 23.7 in men, and ≤ 10.3, 10.4-15.2, 15.3-20.8, and > 20.8 in women. To assess the overall trends of odds ratios across increasing quartiles of total fructose intake, the median fructose intake of each quartile was used as a continuous variable in logistic regression models.

In this study, 45% and 55% of participants were men and women, with mean ages of 40.5 ± 13.6 and 38.6 ± 12.8 years, respectively. Mean dietary intakes of total fructose were 46.5 ± 24.5 g/d (NF: 19.5 ± 10.7 and AF: 26.9 ± 13.9) in men and 37.3 ± 24.2 g/d (NF: 18.6 ± 10.5 and AF: 19 ± 13.7) in women; these intakes were approximately 8 and 7 percent of the total energy intakes, in men and women respectively. Characteristics of the study participants across quartiles of total fructose intakes are presented in Table 1. Participants in the highest quartile of fructose intakes were significantly older as compared with participants in the lowest quartile. Also, means of body mass index and the prevalence of metabolic syndrome were significantly increased across quartiles of dietary intakes of fructose in both sexes. No significant difference in smoking status and physical activity was observed across quartiles of fructose intakes. Age-adjusted means for dietary intakes across categories of dietary fructose are shown in Table 2. Dietary intakes of carbohydrate (% of kcal), total sugar (% of kcal), natural and added fructose (% of kcal) and total dietary fiber (g/d) were significantly increased across quartile of total dietary fructose, whereas dietary fat intakes (% of kcal) were significantly decreased in both sexes. Mean intake of total dietary fructose in the 4^th ^quartile was 2.8 and 3.7 times higher as compared to the first quartile in men and women, respectively. The means of metabolic syndrome features across quartile of fructose intakes are provided in Figure 1. After adjustment for age, energy intake, physical activity and smoking status, the mean of waist circumference across quartiles of fructose intakes were significantly increased (*P *trend = 0.03 and 0.007, in men and women, respectively). Also, dietary fructose intakes in men were positively associated with systolic (*P *trend = 0.02) and diastolic blood pressure (*P *trend = 0.013); in women, this association was observed only for systolic blood pressure (*P *trend = 0.04). There were no significant differences in serum fasting glucose, triglycerides and HDL-C across dietary fructose categories in men and women. The association between total dietary fructose and MetS components is presented in Table 3. Total dietary fructose independently correlated with waist circumference, triglycerides, fasting blood gluocse, systolic and diastolic blood pressure, in both men and women. Compared with those in the lowest quartile of fructose intakes, men and women in the highest quartile, respectively, had 33% (95% CI, 1.15-1.47) and 20% (95% CI, 1.09-1.27) higher risk of the metabolic syndrome, after adjustment for potential confounding variables (Figure 2). Also, men and women in the highest quartile of fructose intakes, respectively, had 39 and 20% higher risk for large waist circumference, 11 and 9% higher risk for hypertension, and both a 9% higher risk of impaired fasting glucose.

**Table 1:** Characteristics of adult participants across quartiles of total dietary intake of fructose: Tehran Lipid and Glucose Study^1^

	Men (*n *= 1141)					Women (*n *= 1396)				
	
	*Q1*	*Q2*	*Q3*	*Q4*	*P *^1^	*Q1*	*Q2*	*Q3*	*Q4*	*p*
Dietary fructose range (g/1000 kcal/d)	≤ 13.3	13.3-17.5	17.6-23.7	> 23.7		≤ 10.3	10.4-15.2	15.3-20.8	> 20.8	
Dietary fructose median (g/1000 kcal/d)	11.1	15.4	19.7	28.1		7.2	12.6	17.9	25.9	
Participants (n)	285	284	285	287		338	359	349	350	
Age (y)	38 ± 12 ^2^	39 ± 13	41 ± 13	43 ± 13	*< 0.01*	37 ± 12	37 ± 12	39 ± 13	41 ± 13	*< 0.01*
Physical activity (MET-h/wk)	40.5 ± 69.2 ^2^	43.4 ± 74.2	45.3 ± 72.8	36.8 ± 62.4	*NS*	29.9 ± 37.9	32.2 ± 41.5	32.3 ± 55.5	32 ± 37.9	*NS*
Body mass index (kg/m^2^)	26.1 ± 0.2 ^3^	26.3 ± 0.2	26.9 ± 0.2	27.3 ± 0.2	*< 0.01*	26.2 ± 0.2	26.8 ± 0.2	27.7 ± 0.2	27.8 ± 0.2	*< 0.05*
Waist/hip ratio	0.94 ± 0.07 ^3^	0.94 ± 0.06	0.95 ± 0.06	0.96 ± 0.05	*< 0.01*	0.81 ± 0.08	0.82 ± 0.09	0.82 ± 0.08	0.84 ± 0.09	*< 0.05*
Total cholesterol (mmol/l)	4.83 ± 0.06 ^3^	4.71 ± 0.06	4.78 ± 0.06	4.81 ± 0.06	*NS*	4.71 ± 0.05	4.73 ± 0.05	4.81 ± 0.05	4.88 ± 0.05	*< 0.05*
LDL-cholesterol (mmol/l)	3.05 ± 0.05 ^3^	2.92 ± 0.05	2.95 ± 0.05	2.99 ± 0.05	*NS*	2.87 ± 0.04	2.89 ± 0.04	2.97 ± 0.04	2.84 ± 0.04	*NS*
Current smoker (%)	24.6	25	23.5	23.7	*NS*	3.0	2.2	2.9	3.4	*NS*
Abdominal obesity (%)	48.4	47.2	52.3	58.5	*< 0.05*	18.6	25.3	27.8	27.4	*< 0.01*
Impaired fasting glucose (%)	12.6	15.1	15.4	18.1	*NS*	10.1	13.9	15.8	14.0	*NS*
Hypertriglyceridemia (%)	44.6	41.2	44.9	46.3	*NS*	27.5	25.6	28.9	34.6	*< 0.05*
Low HDL-cholesterol (%)	58.6	63.3	57.2	64.8	*NS*	69.2	69.6	69.1	69.7	*NS*
Hypertension (%)	21.1	23.6	30.2	32.1	*< 0.01*	12.1	17.3	14.6	19.1	*NS*
Metabolic syndrome (%)	31.9	34.9	37.2	44.3	*< 0.01*	16.3	20.9	21.8	27.1	*< 0.01*

^1^*P *values comparing the characteristics of participants across quartiles of fructose intake using analysis of variance (for age and physical activity), general linear model (for body mass index, waist/hip ratio, total and LDL-cholesterol), and Chi-square test for categorized variables.

^2 ^Values are mean ± SD

^3 ^Values are age-adjusted mean ± SEM

**Table 2:** Dietary intakes of adult participants by quartiles of total fructose intake: Tehran Lipid and Glucose Study

	Males (*n *= 1141)					Females (*n *= 1396)				
	
	*Q1*	*Q2*	*Q3*	*Q4*	*P^2^*	*Q1*	*Q2*	*Q3*	*Q4*	*p*
Energy intake (kcal/d)	2357 ± 41^1^	2538 ± 41	2444 ± 41	2375 ± 41	*< 0.05*	2191 ± 35	2241 ± 34	2237 ± 34	2223 ± 34	*NS*
Carbohydrate (% of energy)	56.7 ± 0.4	58.1 ± 0.4	58.8 ± 0.4	62.4 ± 0.4	*< 0.05*	53.4 ± 0.3	54.9 ± 0.3	56.9 ± 0.3	60.6 ± 0.3	*< 0.05*
Protein (% of energy)	13.6 ± 0.1	13.6 ± 0.1	13.9 ± 0.1	13.8 ± 0.1	*NS*	13.2 ± 0.1	13.8 ± 0.1	13.7 ± 0.1	13.7 ± 0.1	*NS*
Fat (% of energy)	31.1 ± 0.4	30 ± 0.4	29.9 ± 0.4	27.4 ± 0.4	*< 0.001*	34.9 ± 0.4	33.6 ± 0.4	32.3 ± 0.4	29.7 ± 0.4	*< 0.001*
Simple carbohydrate^3 ^(% of energy)	18.9 ± 0.3	21.4 ± 0.3	24.1 ± 0.3	28.6 ± 0.3	*< 0.001*	18.5 ± 0.2	21.5 ± 0.2	23.7 ± 0.2	28.7 ± 0.2	*< 0.001*
Fiber (g/1000 kcal/d)	14.5 ± 0.4	17.3 ± 0.4	17.4 ± 0.4	18.2 ± 0.4	*< 0.001*	14.7 ± 0.3	15.5 ± 0.3	17.3 ± 0.3	18.6 ± 0.3	*< 0.001*
Natural fructose^4 ^(% of energy)	1.7 ± 0.04	2.6 ± 0.04	3.4 ± 0.04	5.2 ± 0.04	*< 0.001*	1.7 ± 0.03	2.6 ± 0.03	3.6 ± 0.03	5.4 ± 0.03	*< 0.001*
Added fructose^5 ^(% of energy)	2.5 ± 0.05	3.6 ± 0.05	4.6 ± 0.05	7.0 ± 0.05	*< 0.001*	1.2 ± 0.05	2.3 ± 0.05	3.6 ± 0.05	5.9 ± 0.03	*< 0.001*
Total dietary fructose										
(% of energy)	4.3 ± 0.09	6.2 ± 0.09	8.0 ± 0.09	12.2 ± 0.09	*< 0.001*	2.9 ± 0.09	5.0 ± 0.09	7.2 ± 0.09	11.3 ± 0.09	*< 0.001*
(% of carbohydrate)	7.7 ± 0.15	10.7 ± 0.15	13.8 ± 0.15	19.6 ± 0.15	*< 0.001*	5.6 ± 0.14	9.3 ± 0.14	12.8 ± 0.14	18.7 ± 0.14	*< 0.001*
(g/d)	26.4 ± 0.7	37 ± 0.7	48.7 ± 0.7	73.6 ± 0.7	*< 0.001*	17.0 ± 0.6	28.2 ± 0.6	40.4 ± 0.6	63.3 ± 0.6	*< 0.001*

^1 ^Data are age-adjusted mean ± SEM

^2 ^*P *values comparing dietary intake of participants across quartiles of fructose intake using linear regression model.

^3 ^Dietary Simple carbohydrates includes sucrose, maltose, galactose, glucose, fructose, maltose.

^4 ^Natural fructose in fructose-containing food such as fruits, vegetables, honey, etc.

^5 ^Fructose content of industrialized foods and beverages containing beet or cane sugar/molasses, corn sweeteners and invert syrup

**Table 3:** Multivariate association between total dietary fructose intake and metabolic syndrome components: Tehran Lipid and Glucose Study

	WC	TG	FBS	HDL-C	SBP	DBP
Men	0.158 (0.001)	0.076 (0.08)	0.115 (0.007)	-0.066 (0.12)	0.103 (0.016)	0.167 (0.001)
Women	0.155 (0.001)	0.130 (0.001)	0.135 (0.001)	-0.012 (0.76)	0.164 (0.001)	0.115 (0.006)

Values are standardized coefficient β (P value) were determined using the linear regression model with adjustment for age, physical activity, body mass index (except for waist circumference and metabolic syndrome), smoking status, energy intake, dietary intake of carbohydrate, fat, simple sugar (except fructose), and fiber. WC; Waist circumference, TG; Triglycerides, FBS; Fasting blood glucose, HDL-C; High density lipoprotein, SBP; Systolic blood pressure, DBP; Diastolic blood pressure.

## Discussion

In this study, the higher intake of dietary fructose was associated with the higher risk of MetS in adults, independent of confounding variables. It should be noted that, in our study the association between dietary fructose and metabolic syndrome and its components was observed only in the third and fourth quartiles of fructose intakes, approximately over 8 and 12% of energy intake (> 50 g/d); while dietary intake of fructose from natural sources including fruits and vegetables, even in the fourth quartile of fructose intakes was only 5% of energy, approximately 30 g/d. Thus, the increased risk of metabolic syndrome and its components may be attributed to increase fructose intake from industrialized foods.

To our knowledge, this is the first study to directly relate dietary fructose intake with the MetS, based on a large population, cross-sectional setting. Johnson and colleagues, in a recent review of animal studies and human clinical trials, proposed that excessive fructose intake, > 50 g/d may be one of the main etiologies of the metabolic syndrome [27]. In the current study, mean intake of total fructose in men and women, was ≈ 8% and ≈ 7% of average energy intake. Dietary fructose from fruit, vegetable and other natural sources, was approximately, 40 and 60% of total fructose in men and women, respectively, while the rest was from beet and cane sugars, soft drink, fruit drink, cookies, candies, jams and bakery products. A recent report from the third National Health Examination Survey (NHANES) demonstrated that over 10% of energy intakes in US adults were from fructose [28].

In this study, dietary fructose intakes > 12% from energy were related to higher chances of having abdominal obesity, and increasing fructose intakes were associated with an increase in body mass index. Findings in line with the results of studies in rats indicating that high fructose diet increased lipogenesis, fat storage, and leptin resistance [29-31]. Administration of 25% fructose diet in overweight and obese subjects for 10 weeks increased intra-abdominal fat storage [7]. Compared with other carbohydrates, mechanisms by which fructose may cause weight gain could be that, fructose increases hunger rating and energy intake, does not stimulate insulin and postprandial leptin levels and inhibits secretion of orexigenic hormone ghrelin, as satiety signals to the brain [32]. Fructose also up regulates de novo lipogenesis in human [7]. The results from epidemiological studies are inconsistent; in a prospective study, consumption of ≥ 1 soft drink per day was associated with increased risk of developing obesity and waist circumference [33]. In another study, this correlation was not found among preschool children [34].

In this study, participants with the highest intakes of fructose had higher risk of impaired fasting glucose. Although fructose does not stimulate insulin secretion, it induced impaired fasting glucose by insulin resistance in the liver and adipose tissue [35,36]. In humans, high doses of fructose decreased insulin sensitivity and glucose tolerance in older, overweight and obese subjects, whereas lower doses did not in young, normal weight men; however fructose caused a 5.5% increase in fasting glucose concentration in these subjects [7,37]. A large cross-sectional study showed that the highest quintile of fructose intake had 13.9% higher C-peptide levels, as a risk factor for insulin resistance [38].

In the present study, fructose intakes > 72 and 63 g/d in men and women were associated with 11 and 9% increased risk of hypertension, respectively. In one study, administration of 200 g/d fructose in healthy adults significantly increased systolic and diastolic blood pressure [39]. In the Framingham Heart Study, consumption of soft drinks increased risk of hypertension [33]. Furthermore, recent data from a cross-sectional study demonstrated that high fructose intake in the form of added sugar was associated with higher blood pressure levels in adults [40]. Some studies proposed that hypertension-induced effects of fructose may be related to the ability of fructose to increase serum uric acid and enhance reabsorption of salt and water in the small intestine and kidney [41,42]. However in a recent cross-sectional study, no association was found between dietary fructose and risk of hyperuricemia in adults [21]. Also, blood pressure did not change with the high consumption of fructose over the course of the 10 week intervention period [7].

The current study showed that there were no significant associations between fructose intake and fasting serum triglycerides, and HDL-cholesterol. Excessive fructose intake for 2 weeks in healthy subjects resulted in hypertriglyceridemia and decreased HDL-cholesterol levels ^(39)^. Stanhope et al showed that administration of a diet with 25% fructose for 10 weeks did not change fasting TG concentrations, but increased postprandial de novo lipogenesis and TG concentrations, fasting and postprandial apo B and LDL-C concentrations in overweight and obese subjects [7]. However results from a recent meta-analysis, suggested that dietary fructose intakes less than 50 g/d had no adverse effect on fasting and postprandial triglycerides [43]. Although, in an observational study of overweight children, no association was observed between fructose consumption and HDL, LDL, total cholesterol and triglyceride concentrations, fructose intake was a significant predictor of LDL particle size as an atherogenic risk factor [44]. More observational studies need to be conducted on the association between usual intakes of fructose and lipid profiles among adults.

One limitation of this study is the use of an FFQ for collecting dietary data. Also total fructose intake was estimated approximately, because of a lack of fructose content data for many food items. In addition, this is a cross sectional study which limits ability to determine causality between dietary fructose intake, MetS and its components.

In conclusion, in this study, higher dietary fructose intake was significantly associated with the increased risk of metabolic syndrome and some of its risk factors including abdominal obesity, hypertension and impaired fasting glucose in adults, even after adjustment for demographic, anthropometric and dietary intakes.

## Competing interests

The authors declare that they have no competing interests.

## Authors' contributions

The project idea for this study was from FHE. The project was design by FHE, P.M and ZB. The ZB, FH and SHN analyzed and interpreted the data. ZB, PM and FA prepared the manuscript. All authors read and approved the final version of the manuscript to be submitted.
